# Metabolic Profile of Whole Unstimulated Saliva in Patients with Sjögren’s Syndrome

**DOI:** 10.3390/metabo13030348

**Published:** 2023-02-27

**Authors:** Giacomo Setti, Valeria Righi, Adele Mucci, Lucia Panari, Giuditta Bernardelli, Elisabetta Tarentini, Anna Gambini, Ugo Consolo, Luigi Generali, Cristina Magnoni, Marco Meleti, Gilda Sandri, Pierantonio Bellini

**Affiliations:** 1 Dentistry and Oral-Maxillofacial Surgery Unit, University of Modena and Reggio Emilia, Largo del Pozzo 71, 41125 Modena, Italy; 2 Department for Life Quality Studies, University of Bologna, Corso d’Augusto 237, 47921 Rimini, Italy; 3 Department of Chemical and Geological Sciences, University of Modena and Reggio Emilia, Via G. Campi 103, 41125 Modena, Italy; 4 Pathology Unit, Azienda USL-IRCCS di Reggio Emilia, Viale Risorgimento 80, 42123 Reggio Emilia, Italy; 5 Dermatology Unit, University of Modena and Reggio Emilia, Largo del Pozzo 71, 41125 Modena, Italy; 6 Centro Universitario Odontoiatria, University of Parma, Via Gramsci 14, 43126 Parma, Italy; 7 Rheumatology Unit, University of Modena and Reggio Emilia, Largo del Pozzo 71, 41125 Modena, Italy

**Keywords:** Sjögren’s Syndrome, metabolomics, nuclear magnetic resonance, saliva

## Abstract

Primary Sjögren’s Syndrome (pSS) is a multi-system autoimmune disease that involves the exocrine glands. Lymphocytes infiltrate the gland tissue, leading to anatomical modification and hypofunction. Even if the prognosis of pSS is favorable, quality of life is typically reduced due to the diverse manifestations of the disease. The aim of this study is to compare the salivary metabolomes of pSS with healthy controls (HCs). Seven cases were selected from a cohort of pSS patients, and six age- and sex-matched HCs were recruited from a cohort of volunteers. Whole unstimulated saliva was collected for NMR analysis. Our metabolomic analysis focused on 360 ms total echo 1D ^1^H NMR CPMG spectra. Metabolites detected with CPMG NMR spectra were assigned through 2D NMR spectra (COSY, TOCSY, and HSQC). About 50 metabolites were detected and assigned. Unsupervised exploratory PCA returned partial clustering, and PLS-DA improved the separation between pSS and HCs, highlighting a pool of metabolites distinctly describing each group. Despite the limited number of samples, the presented preliminary data are promising. PLS-DA indicated well-defined group separation, suggesting that the application of ^1^H-NMR metabolomics is suitable for the study of pSS.

## 1. Introduction

Primary Sjögren’s Syndrome (pSS) is a chronic, slowly progressing autoimmune disease characterized by xerostomia and keratoconjunctivitis sicca as clinical hallmarks. pSS may evolve from organ-specific autoimmune exocrinopathy to systemic disease.

pSS’s prevalence is widely described as fewer than 50 cases per 100,000 inhabitants. Women are six to nine times more likely to be affected than men, and the typical age at diagnosis is around 60–70 years [1].

Distinctive recurrent phases are associated with the pathogenesis of pSS: (1) environmental stimuli (e.g., viral infections) act to trigger a certain condition involving genetic predisposition, the presence of epigenetic factors, and hormonal regulation; (2) overturn of normal exocrine gland function (e.g., salivary, lacrimal, and diffuse glandular tissue of the esophagus, stomach, bowel, pancreas, and bladder) caused by alteration of epithelial cell activity [2]; and (3) B and T lymphocytes become hyperactive, leading to glandular tissue infiltration. Consequently, B-cells intensify auto-antibody production. The chronic inflammation in pSS worsens the glandular structure modification and contributes to hypofunction [3].

The following clinical manifestations are consequences of systemic involvement: dryness secondary to exocrinopathy, autoimmune epithelitis with periepithelial lymphocytic infiltration of target organs, associated organ-specific autoimmunity with specific autoantibodies, the presence of immune complexes or cryoglobulinemia, and clonal lymphocytic expansion [4]. During the natural course of the disease, 75% of patients will develop at least one extraglandular manifestation, which can occur at diagnosis or during follow-up. In general, the manifestations caused by lymphocytic infiltration of a target organ are indolent and durable (e.g., sicca syndrome, renal tubular acidosis, pulmonary involvement) while the autoimmune disorders linked to immune complexes or autoantibodies have a more unpredictable course, with flares and remissions. Overall, quality of life is strongly reduced due to the diverse manifestations of the disease. pSS can be disabling and associated with significant functional status impairment related to oral and/or ocular dryness, systemic activity, pain, fatigue and daytime somnolence, anxiety, psychological distress, and depression symptoms [5].

The progression of pSS is favorable, with the patient’s life expectancy being comparable to that of the general population. However, a sub-group of patients will have unfavorable prognoses. Specific risk factors are identifiable, such as advanced age, atypical parotid gland imaging (e.g., scintigraphy), extraglandular involvement, vasculitis, anti-SSB/Ro positivity, reduction in serum complement components (e.g., C3, C4, and CH50 inflammatory protein), and cryoglobulinemia.

The excess mortality is generally attributed to the development of non-Hodgkin’s lymphoma, solid tumors, or uncommon (but severe) visceral involvement, such as cardiovascular disease and infections [6]. In spite of the predominantly female involvement, evidence of a higher incidence of lymphoma and interstitial lung disease in males with pSS has been reported, suggesting a sex-specific preference for some clinical manifestations [7].

There is still no single clinical, laboratory, pathological, or radiological feature that could serve as a gold standard for the diagnosis and/or classification of this syndrome [8]. Diagnostic criteria are provided in the 2016 ACR/EULAR Consensus of Classification Criteria for pSS, which are based on the sum of weighted scores applied to five items: anti-SSA/Ro antibody positivity, a focus score of ≥1 foci/4 mm^2^, an ocular staining score ≥5 (or van Bijsterveld score ≥ 4) in at least one eye, a Schirmer’s test result of ≤5 mm/5 min, and an unstimulated salivary flow rate (SFR) of ≤0.1 mL/minute [9].

The chemical–physical properties and volume of whole saliva (WS) can grossly vary among people, as well as in the same person, according to endogenous and exogenous factors (e.g., age, gender, circadian rhythm, psychological state, nutrition, diseases, drugs, and environmental exposures). Moreover, qualitative variations of saliva related to the presence and concentration of specific categories of molecules have been described [10,11]. Additionally, saliva has recently been widely investigated in order to understand its role in potential systemic disease and cancer diagnosis [12,13].

As the salivary glands are a major site of autoimmune destruction in pSS, changes in salivary components may reflect the pathogenesis of the disease. Taking advantage of emerging ‘omics’ technologies, saliva has been explored for the discovery of biomarkers for the diagnosis and prognosis of pSS [8].

More than a few proteomic studies have shown differential protein expression in the saliva of pSS patients and healthy control subjects, which could lead to the identification of potential biomarkers [14]. In addition, transcriptome analysis of the saliva from pSS patients and controls revealed differences in mRNA expression levels [15].

Metabolomics has been successfully proposed to characterize pSS saliva, compared with that of healthy controls (HCs). Saliva from patients with pSS has been studied using different mass spectrometry techniques: gas chromatography–mass spectrometry (GC–MS) [16] and ultra-performance liquid chromatography–mass spectrometry (UPLC–HRMS) [17].

Some other studies report the use of Nuclear Magnetic Resonance (NMR) spectroscopy on salivary samples [18,19] for pSS investigation.

It has been shown that NMR is a powerful and reproducible technique for metabolic profiling and, when combined with multivariate analysis, it can be used as a biomarker diagnostic tool [20]. Mikkonen et al. have investigated the salivary metabolic profile of patients with pSS. They identified 24 metabolites in samples of stimulated saliva; metabolites such as choline, butyrate, proline, taurine, alanine, phenylalanine, and glycine have been shown to have significantly higher concentrations in saliva from pSS patients than HCs [19]. Moreover, Herrala et al. described 21 variables that emerged from the inter- and intra-individual evaluation of salivary samples collected at different time points and compared with HCs. Levels and outputs of some peculiar metabolites, such as choline, taurine, alanine, and glycine significantly distinguished cases from controls, suggesting their potential application for pSS diagnosis and follow-up [18].

The use of NMR spectroscopy has several advantages. The technique does not require pre-treatment of the sample and the amount of sample needed is limited, which is significant in our study [21]. The NMR analysis does not exceed half an hour and allows for the simultaneous detection of small and large metabolites in the sample. 2D NMR experiments allow the assignment of new metabolites, even in the absence of a standard, with a skeleton reconstruction process described elsewhere [22].

Finally, NMR spectroscopy allows unbiased quantification and greater reproducibility of data.

The aim of this study is to improve the NMR database of metabolites on pSS saliva and to compare the salivary metabolome of pSS with HCs through the application of untargeted metabolomics performed with ^1^H-NMR.

## 2. Materials and Methods

### 2.1. Population selection

The evaluated subjects were part of a single-center case-control study (Ethical Committee Code: 1183/2018/SPER/AOUMO).

Between 2011 and 2017, 124 patients received a biopsy of the minor salivary glands due to suspicion of SS. Related histological slides were re-assessed to standardize the diagnostic criteria [9,23]. All patients were in follow-up with the rheumatology service of University of Modena and Reggio Emilia at the time of review of specimens. These results were matched with data collected during clinical and serological evaluations.

A total of 60 diagnoses were confirmed for pSS, which constituted the cohort for further sample selection.

Strict selection criteria were applied in order to obtain the most homogeneous study population. Patients with a history of head and neck radiotherapy, HIV or HCV infection, lymphoma, sarcoidosis, graft versus host disease, and use of anticholinergic drugs were excluded. Furthermore, the presence of oral lesions, active oral infections, diabetes, smoking habits, and oncological diseases also constituted exclusion criteria. Unstimulated whole salivary flow was assessed under the following standardized conditions: the subject was advised to refrain from intake of any food or beverage (except for water) as well as from smoking one hour before the test session; immediately before collection, patients rinsed their mouth with water for 1 min; and patients passively drooled or spat into a measuring container over 15 min [24].

After the application of exclusion criteria, between January and July 2019, 7 female cases were selected for salivary investigation (mean age 65.4). In addition, 6 sex- and age-matched HCs were selected from a cohort of volunteers.

The serological status of patients was also considered, to evaluate the level of anti-SSA/Ro, anti-SSB/La, rheumatoid factor (RF), and antinuclear antibody (ANA). Moreover, clinical information such as referred symptoms and other test results were considered (e.g., Schirmer test). The characteristics of the patients are summarized in Table 1.

### 2.2. Saliva Collection

A thorough oral evaluation was performed to determine potential pathological conditions. Saliva samples were collected using standard techniques, according to Navazesh [25]. All recruited patients were asked not to eat, drink, smoke, or use oral hygiene products one hour before saliva collection. Immediately before collection, patients rinsed their mouths with water for 1 min. Samples from the two groups were collected in the morning (9:00 to 12:00) to minimize the influence of the circadian rhythm on salivary composition. Unstimulated whole saliva was passively collected or spat into a sterile Eppendorf cuvette, which was kept on ice during the entire process. All samples were immediately frozen in liquid nitrogen for transportation and stored in a refrigerator at −80 °C until further analysis. Specimen anonymization took place at this stage.

### 2.3. Sample Preparation

Each frozen saliva sample was thawed at room temperature and centrifuged at 15,000× *g* rpm for 10 min at 4 °C to remove cells, cellular debris, and mucins, according to Gardner et al. [26]. To equalize the pH measurements, all samples were buffered with a solution containing trisodium phosphate (TSP), 500 μL of supernatant was mixed with 100 μL of TSP buffer (pH 7.45), and 500 μL of this solution was used for NMR.

### 2.4. NMR Data Collection and Analysis

**Figure 1:**
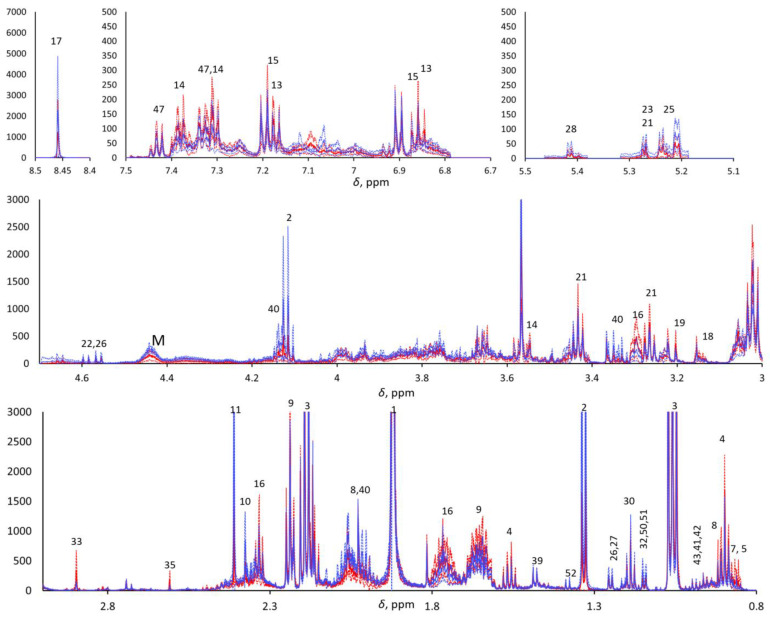
Five enlarged regions of the mean pSS (continuous red line) and mean HC (continuous blue line). ^1^H CPMG NMR spectra (±standard deviations, dotted lined; red and blue for pSS and HCs, respectively). M indicates macromolecules.

The ^1^H-NMR metabolomics was blindly performed, as the operator was not aware of the provenance of the samples. An NMR Bruker Avance III HD 600 MHz spectrometer was used for data acquisition. One- (^1^H) and two-dimensional experiments—e.g., homonuclear ^1^H,^1^H correlation spectroscopy (COSY), total correlation spectroscopy (TOCSY), and ^1^H,^13^C heteronuclear single quantum coherence (HSQC)—were performed in order to identify the metabolites. The 1D ^1^H-NMR Carr–Purcell–Meiboom–Gill (CPMG) pulse sequence was used to highlight the narrow signals of metabolites and attenuate the broad signals of macromolecules. Due to the aqueous nature of saliva, pre-saturation was applied to suppress the water signal. The NMR spectra were pre-processed as previously reported [27]. In brief, the ^1^H CPMG NMR spectra were acquired with 4 s water pre-saturation, 2.27 s acquisition time, 106k data points, 40 ppm spectral width, and total echo time of 360 ms (2 ms single echo) 256 scans. They were zero-filled to 128 k and processed (TopSpin ^®^ 4.0.7, Bruker Corp., Billerica, MA, USA) with a 0.5 Hz exponential line broadening, then manually phased and baseline corrected. The residual water signal was cut, and the spectra were aligned, binned (0.001 ppm), and normalized with respect to the total area in the range of 0.8–8.5 ppm (MNova, Mestrelab Research, S.L., Santiago de Compostela, Spain; Metaboanalyst 4.0, XiaLab, McGill University, Montreal, QC, Canada) to 7706 final spectral points. The chemical shift scale was calibrated with respect to the Me_3_Si signal of trimethylsilylpropanoic acid (TSP) at 0 ppm. The areas of selected signals from 30 metabolites were estimated by deconvolution through the MNova Line Fitting routine. Each spectrum was normalized with respect to its total spectral area (after cutting the residual water signal) prior to computing the point-by-point mean spectra for the two classes and their standard deviations (Figure 1).

### 2.5. Statistical Analysis

Multivariate statistical analysis was conducted on binned and normalized spectra (7706 final spectral points; see Section 2.4) to analyze the spectral profiles between 0.8 and 8.5 ppm, after Pareto scaling, using the MetaboAnalyst 5.0 program, a free web-based metabolomics data analysis software [28]. Both principal component analysis (PCA) and partial least-squares discriminant analysis (PLS-DA) were performed for class discrimination.

Deconvoluted areas of selected peaks from ^1^H CPMG HR-MAS NMR spectra were obtained with an automated controlled fitting routine implemented in MNova (Mestrelab; Metaboanalyst 4.0, XiaLab McGill). The paired two-sample Student’s t-test was applied to determine the mean difference between two sets. A *p*-value < 0.05 was considered statistically significant. Fold-change analysis was carried out on deconvoluted signals.

## 3. Results

### Metabolomic and Statistical Analysis

The metabolomic analysis focused on 360 ms total echo 1D ^1^H NMR CPMG spectra. Metabolites detected with CPMG NMR spectra were assigned through 2D NMR spectra (COSY, TOCSY, and HSQC), and approximately 50 metabolites were detected and assigned (see Supplementary Table S1). ^1^H CPMG NMR spectra evidenced relevant variations in overall metabolite concentration among the saliva samples within each class, and both PCA and PLS-DA did not show any significant trend when applied to the row data with and without Pareto scaling. To overcome this problem, we compared the spectra after normalization with their total area, excluding the residual water signal. Visual comparison of the average pSS and HC ^1^H CPMG NMR spectra of the two classes (Figure 1) highlighted the metabolites that were more abundant in pSS samples (e.g., 4-hydroxyphenylacetic acid, phenylacetic acid, δ-valerolactam, taurine, choline, ethanolamine, trimethylamine, methylamine, 5-aminopentanoic acid, butyric acid, and other small organic acids) and in HC samples (e.g., formic acid, monosaccharides, macromolecules, proline, and lactic acid), although the t-test confirmed statistically significant differences (*p* < 0.05) only for methylamine (higher in SS), proline (higher in HCs), and residual signals from macromolecules between 4.2 and 4.5 ppm (higher in HCs). All other differences between the two classes can be considered only as trends.

**Figure 2:**
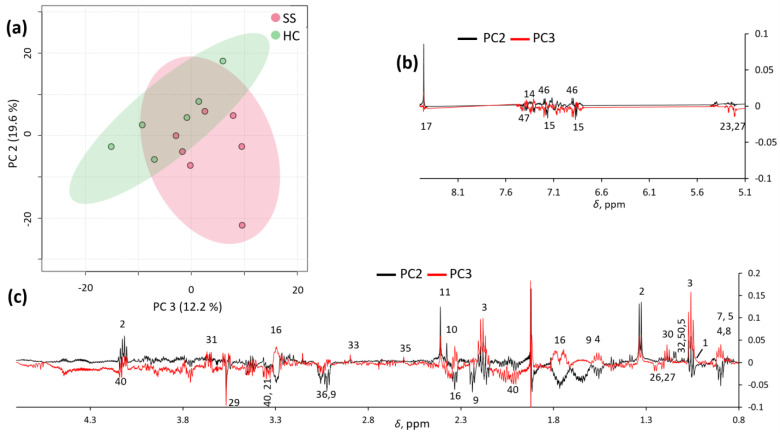
PC2/PC3 score plot (**a**) and loading profiles (**b**,**c**) obtained by PCA on spectra.

Unsupervised exploratory PCA was also conducted on the spectra, and partial clustering was observed, especially in the PC2/PC3 score plot (Figure 2a). These two principal components explained 19.6% and 12.2% of the total variance, respectively. Inspection of the PC2 and PC3 loadings (Figure 2b) confirmed that pSS samples were characterized by a number of metabolites (as reported in Supplementary Table S1), which potentially allow for their discrimination from HC samples.

**Figure 3:**
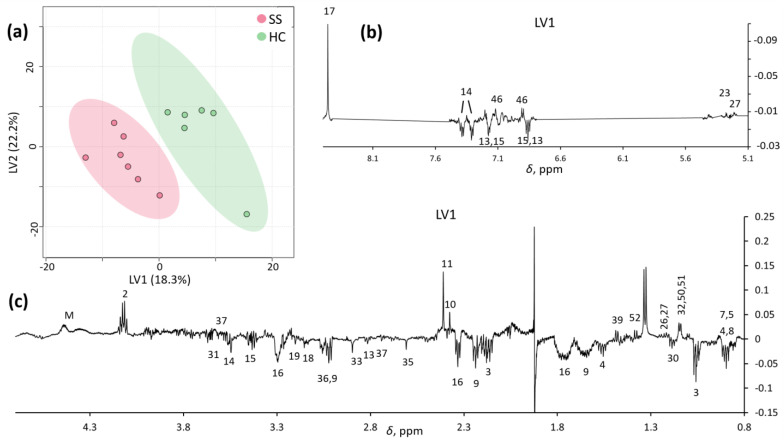
LV1/LV2 score plot (**a**) and loading profiles (**b**,**c**) obtained by PLS-DA on spectra.

PLS-DA analysis improved the separation between SS and HCs in the first two latent variables, LV1 and LV2 (Figure 3a). The LV1 loading profile (Figure 3b,c) confirmed a pool of metabolites distinctly describing each group, most of which were the same as those obtained by PCA (Table 2).

Finally, a total of 30 metabolite signals, selected according to their low overlap with the neighboring signals, were deconvoluted from the 1D ^1^H-NMR CPMG NMR spectra and analyzed by PCA, PLS-DA, t-test, and fold-change analyses (Table 3). PCA and PLS-DA did not indicate any improvement with respect to the spectral analysis. Only methylamine was found to significantly differ (*p* < 0.05) between the two groups by the t-test, both when considering absolute signal areas (in analogy to the approach of Mikkonen et al.) and signal areas normalized with respect to the corresponding total spectral area (parallel to what we performed on spectra). The fold-change analysis was more straightforward and pointed to the subset of metabolites already evidenced by PCA and PLS-DA.

## 4. Discussion

Metabolomics has been successfully proposed to facilitate the characterization of pSS saliva compared with healthy controls (HCs); however, the limited number of published studies has led to a lack of evidence regarding the reliability of metabolomics as a diagnostic tool.

Different analytical approaches have been proposed, such as nuclear magnetic resonance (NMR) and mass spectrometry (MS). The wide application of MS technology is a consequence of its high sensitivity, which permits qualitative exploration of the salivary metabolome, even when collecting a limited number of samples. The sensitivity of MS allows for the detection of many metabolites (e.g., from 300+ to 1000+ if GC-MS or LC-MS is performed), the platform costs are cheap, and it is ideal for targeted metabolomics. However, the quantitative description of selected variables requires an additional platform.

Similar data acquisition can be obtained by NMR, which is less sensitive than MS, but the obtained data are simultaneously qualitative and quantitative. NMR data are more reproducible than MS data, the acquisition execution is faster, minimal sample preparation is required, and tissue or matrix extraction does not need to be performed. Therefore, the average cost per sample is cheaper for NMR than for MS.

Through untargeted ^1^H-NMR, Mikkonen et al. identified 24 metabolites in stimulated whole saliva, with significant variations of specific molecules between analysis groups. The concentration of choline and taurine was significantly higher (*p* < 0.001) in the pSS patients compared with that of the healthy controls. Moreover, alanine and glycine were significantly higher (*p* = 0.004, *p* = 0.007, respectively) in concentration in the pSS group. Butyrate (*p* = 0.034), phenylalanine (*p* = 0.026), and proline (*p* = 0.032) were only slightly higher in pSS saliva samples than in those of the controls. Notably, a strong relationship between the salivary flow change and two metabolites concentration emerged; amino-acids choline and taurine concentrations showed a significantly increase in cases with reduced salivation (*p* = 0.0001 and *p* = 0.0006 respectively) [19].

Herrala et al. detected 24 variables in salivary samples analyzed by NMR. A further investigation was conducted on 21 selected metabolites at different time points, making both an intra- and inter-individual comparison; based on previous results, the most significantly differing metabolites (pSS vs. HCs), such as choline, taurine, alanine, and glycine, were chosen for in-depth analysis. All the variables were able to distinguish the two groups almost at any time point [18].

Kageyama et al. analyzed the metabolite profiles of whole saliva from 14 pSS patients with GC-MS. Of the 88 metabolites identified, 41 were significantly decreased in pSS patients compared with HCs, most likely reflecting salivary gland damage; in particular, glycine, tyrosine, uric acid, and fucose contributed to the loss of biodiversity in the pSS versus HCs salivary samples [16].

Li Z. et al. performed UPLC-HRMS to identify 38 metabolites, mostly amino acids, with potential diagnostic value. Their results showed that phenylalanine, tyrosine, tryptophan, and proline pathways are upregulated in pSS patients [17].

Metabolomic analysis of our ^1^H CPMG NMR spectra indicated that the pSS group salivary metabolome was characterized by higher relative amounts of amines (methylamines, putrescine, and sarcosine), organic acids (mainly belonging to propanoic, butanoic, pentanoic, phenyl acetic, and phenylpropanoic series), and 5-aminopentanoic acid and its lactam (δ-valerolactam). The HC group, in contrast, was characterized by higher relative amounts of lactic, succinic, pyruvic, and formic acids, as well as amino acids.

Our results show a reduction in fucose and amino acids (e.g., glycine and tyrosine) in the saliva of pSS cases. These data are coherent with those of Kageyama et al., determined by GC-MS [16]. However, glycine concentration seems to have a countertrend when compared with NMR results of Mikkonen et al. and Herrala et al., which both report significantly increasing salivary concentration in pSS compared with HCs [18,19].

Our NMR results show that butyrate was higher in pSS patients than in HCs, but proline was higher in HCs than in pSS patients; thus, we partially agree with results of Mikkonen et al. that found butyrate was slightly elevated in the pSS population vs. HC, as well as the amino acid proline [19].

The higher concentration of proline in HC saliva is likely representative of normal salivary physiology, as reported by Meleti et al.; salivary mucins—a heterogeneous group of glycoproteins synthesized and secreted by the submandibular, sublingual, and minor salivary glands—contribute to the salivary concentration of proline. This amino acid was found to be one of the most concentrated in unstimulated whole saliva of healthy subjects [11]. Especially, the proline-rich proteins (PRPs) are a huge family of salivary proteins produced by major glands, representing nearly 70% of the total protein in human saliva [29]. Proline accounts for about 25–40% of the amino acid content of PRPs [30], which are encoded by six genes [31].

Methylamine is a monoalkylamine that occurs endogenously from amine catabolism. Its tissue levels are altered under some pathological conditions, including diabetes and inflammatory bowel diseases [32]. The statistically significant concentration of methylamine in the salivary samples of the pSS group (*p* < 0.05) can be linked to bacterial metabolism. It can be speculated that alteration of the salivary qualitative composition affects its buffer potential and the capability of the fluid to interact with microbial populations.

The serological status of pSS patients was positive for anti-SSA/Ro in five cases out of seven (SJ02, SJ03, SJ05, SJ06, and SJ07). Both SJ01 and SJ04 tested negative for anti-SSA/Ro but positive for RF (SJ01) and ANA (SJ04). In addition, these cases were diagnosed with pSS according to symptoms, clinical testing, and histopathology. Despite the reported symptoms of xerostomia in six out of seven pSS patients, the salivary flow rate test was positive for hyposalivation in only two cases [9]. In such patients, one with gland hypofunction did not report xerostomia as a symptom. A possible explanation for this finding may reside in the test interpretation itself. Even if ACR/EULAR guidelines set the test limit to <0.1 mL/min and this value is considered for disease management and therapy administration [33], recently published reviews have suggested an adjustment of the threshold for hyposalivation, modifying the cutoff to <0.2 mL/min to better fit the population over 60 years of age, thus increasing the sensitivity and specificity of the pSS diagnosis [34,35].

The main limitation of this preliminary study was the small sample size, allowing for limited statistical interpretation. A wider population will be required to observe better separation of groups in the PCA analysis. Nevertheless, only PLS-DA can provide good insight. Moreover, in this preliminary phase, no clustering was obtained when deconvolution was attempted. Another pitfall of this study was the assessment of oral hygienical status prior to salivary collection. Considering the oral cavity is an “open” environment, whole saliva composition is affected by the presence of bacteria [36]. It is worth mentioning that we found a change in the concentration of three metabolites of bacterial origin (acetate, propionate, and trimethylamine) in pSS compared with HC saliva. We interpreted the presence of these metabolites as an indication of microbial contamination due to plaque, as has been reported by Aimetti et al. [37,38] and Chen et al. [39] Furthermore, emerging results have indicated that acetate, propionate, trimethylamine, and butyrate are increased in the NMR salivary metabolome of chronic periodontitis patients [37], as well as under severe gingivitis conditions [40].

Notwithstanding such limitations, the presented preliminary data are promising.

We were able to distinguish the pSS group from the control group, and these results confirmed that ^1^H NMR spectroscopy-based salivary metabolomics is appropriate for screening and monitoring pSS. Stronger evidence and further experiments are required to consolidate the diagnostic and prognostic potential of salivary metabolomics with respect to Sjögren’s syndrome.

## Figures and Tables

**Table 1:** Histological, serological, and clinical characterization of enrolled patients.

Patient	Histological Assessment before Standardization	Focus Score Following Slides Review [23]	Antibodies	Unstimulated SFR Test for Hyposalivation	Schirmer Test	Reported Symptoms
SJ01	1 focus	1, severe	anti-SSA− anti-SSB− ANA− RF+	negative	negative	xerophthalmia, xerostomia
SJ02	grade 1	3, severe	anti-SSA+ anti-SSB− ANA− RF−	negative	negative	xerophthalmia, xerostomia
SJ03	focus score 1+	5, severe	anti-SSA+ anti-SSB− ANA− RF−	positive	positive	xerophthalmia, xerostomia
SJ04	7 clusters, grade 2+	5, severe	anti-SSA− anti-SSB− ANA+ RF−	negative	negative	xerophthalmia, xerostomia
SJ05	focus score > 1, grade 4	5, severe	anti-SSA+ anti-SSB− ANA− RF−	negative	negative	xerophthalmia, xerostomia
SJ06	grade 1	1, severe	anti-SSA+ anti-SSB− ANA+ RF−	negative	negative	xerophthalmia, xerostomia
SJ07	1 focus	1, severe	anti-SSA+ anti-SSB+ ANA− RF−	positive	positive	NR

ANA, antinuclear antibodies; RF, rheumatoid factor; NR, not reported.

**Table 2:** Most significant metabolites in each group (red, pSS; green, HCs) obtained by PCA and PLS-DA from ^1^H-NMR CPMG NMR spectra.

PCA		PLS-DA	
Sjögren’s Syndrome	Healthy Controls	Sjögren’s Syndrome	Healthy Controls
2-methylbutanoic acid	lactic acid	2-methylbutanoic acid	lactic acid
4-methylpentanoic acid	succinic acid	4-methylpentanoic acid	succinic acid
3-methylbutanoic acid	pyruvic acid	3-methylbutanoic acid	pyruvic acid
2-methylpropionicacid	formic acid	2-methylpropionicacid	formic acid
butanoic acid	propylene glycol or unknown 50/51	butanoic acid	propylene glycol or unknown 50/51
propanoic acid	fucose	propanoic acid	fucose
ethanol	galactose	ethanol	galactose
glycerol	proline	glycerol	tyrosine
5-aminopentanoic c	glycine	5-aminopentanoic c	alanine
δ-valerolactam	taurine	δ-valerolactam	unknown 52
ethanolamine	phenylalanine	choline	
methylamines		ethanolamine	
putrescine		methylamines	
phenylacetic acid		putrescine	
4-hydroxyphenylacetic acid		sarcosine	
		phenylacetic acid	
		4-hydroxyphenylacetic acid	
		phloretic acid	

**Table 3:** Results of t-test (*p* value) and fold-change (FC SS/HC) analyses of deconvoluted signals of SS and HC samples. Metabolites highlighted in red were higher in pSS, those in green were higher in HCs.

Metabolite	Deconvoluted Signal (ppm)	*p*-Value *	FC *	*p*-Value ^$^	FC ^$^
2-methylbutanoic acid	0.86	0.12	2.9	0.07	2.3
4-methylpentanoic acid	0.88	0.23	2.0	0.31	2.0
butanoic acid	0.90	0.29	2.2	0.10	1.5
3-methylbutanoic acid	0.91	0.21	2.5	0.09	2.0
1.15 ppm	1.15	0.24	0.5	0.05	0.5
ethanol	1.19	0.14	1.5	0.43	1.3
fucose	1.25	0.59	0.9	0.26	0.7
lactic acid	1.33	0.42	0.4	0.39	0.5
alanine	1.48	0.71	0.9	0.28	0.8
acetic acid	1.92	0.66	1.2	0.26	1.1
propanoic acid	2.19	0.88	1.1	0.56	1.1
5-aminopentanoic acid	2.24	0.37	1.9	0.23	1.3
δ-valerolactam	2.33	0.38	3.2	0.24	1.8
pyruvic acid	2.38	0.34	0.5	0.33	0.7
succinic acid	2.41	0.38	0.3	0.35	0.5
methylamine	2.61	0.01	2.3	0.05	2.3
dimethylamine	2.73	0.90	1.0	1.00	1.0
sarcosine	2.74	0.96	1.0	0.98	1.0
trimethylamine	2.90	0.08	3.0	0.13	2.7
ethanolamine	3.15	0.73	0.9	0.58	1.2
unknown 53	3.16	0.35	2.0	0.41	1.4
taurine	3.26	0.35	1.4	0.15	1.3
proline	3.34	0.56	0.7	0.07	0.5
phenylacetic acid	3.54	0.42	2.4	0.35	1.6
glycine	3.56	0.72	1.3	0.67	0.8
phloretic acid	6.85	0.14	4.0	0.07	3.1
4-hydroxyphenylacetic acid	6.87	0.65	1.4	0.85	1.1
tyrosine	6.90	0.67	0.8	0.64	0.9
phenylalanine	7.42	0.64	1.2	0.38	1.3
formate	8.46	0.57	0.6	0.47	0.6

* Absolute areas of the signals. ^$^ Areas of the signals normalized with respect to the total spectral areas.

## Data Availability

No new data were generated.

## Supplementary Materials

The following supporting information can be downloaded at: https://www.mdpi.com/article/10.3390/metabo13030348/s1, Table S1: ^1^H NMR chemical shift assignments of the metabolites detected in saliva.
