# Supplementary material for: Metabolic Profile of Whole Unstimulated Saliva in Patients with Sjögren’s Syndrome
Source: Metabolites. 2023 Feb 27;13(3):348. doi: 10.3390/metabo13030348 (PMC10054882; doi:10.3390/metabo13030348)
Supplement: Supplementary file 1 [file metabolites-13-00348-s001.zip › metabolites-2232783-supplementary.pdf]

Supplementary Table. ^{1}H NMR chemical shift assignments of the metabolites detected in saliva.

	Metabolites	ppm	a		Metabolites	ppm	a		Metabolites	ppm	a
1	acetic acid	1.92	s	*19*	choline	3.20	s	*37*	sarcosine	2.74<br>3.61	s<br>s
2	lactic acid	1.33<br>4.12	d<br>q	*20*	phosphocholine	3.22	s	*38*	threonine	1.33<br>4.26<br>3.59	d
3	propanoic acid	1.06<br>2.19	t<br>q	*21*	taurine	3.26<br>3.43	t<br>t	*39*	alanine	1.48<br>3.79	d<br>q
4	butanoic acid	0.90<br>1.56<br>2.16	t<br>sx<br>t	*22*	β-galactose	4.59<br>3.50<br>3.66<br>3.94	d<br>t<br>d<br>d<br>d	*40*	proline	2.02<br>2.07;<br>2.36<br>3.34;<br>3.42<br>4.13	m<br>m<br><br>dt<br><br>dd
5	2-methylbutanoic acid	0.86<br>1.39,<br>1.49<br>1.05<br>2.20	t<br>m<br>d	*23*	α-galactose	5.27<br>3.81<br>3.86<br>4.00	d<br>d<br>d<br>d<br>d<br>d	*41*	valine	0.99<br>1.05<br>2.27<br>3.62	d<br>d<br>m<br>d
6	2-methylpropionic (isobutyric acid)	2.39<br>1.07	sp<br>d	*24*	β-glucose	4.65<br>3.25<br>3.49<br>3.42	d<br>t	*42*	leucine	0.96<br>0.97<br>1.72<br>3.73	d<br>d<br><br>d
7	4-methylpentanoic (isocaproic acid)	0.88<br>1.52<br>1.45<br>2.19	d<br><br>q<br>t	*25*	α-glucose	5.24<br>3.54	d	*43*	isoleucine	0.94<br>1.01<br>1.48<br>1.98<br>3.67	t<br>d
8	3-methylbutanoic acid (isovaleric acid)	0.91<br>1.96<br>2.05	d<br>m<br>d	*26*	β-fucose	4.56<br>3.45<br>3.65<br>3.76<br>3.81<br>1.25	d<br>t<br>d<br>d<br>d<br>q<br>d	*44*	lysine	3.76<br>1.90<br>1.45<br>1.73<br>3.03	
9	5-aminopentanoic acid	2.24<br>1.64<br>1.68<br>3.02	t<br>m<br>m<br>t	*27*	α-fucose	5.21<br>3.77<br>3.86<br>3.82<br>4.20<br>1,21	d<br>d<br>d<br>d<br>d<br>d<br>q<br>d	*45*	ornithine?	3.78<br>1.94<br>3.06	
10	pyruvic acid	2.38	s	*28*	maltose?	5.42<br>5.23?<br>4.66	d<br>d<br>d	*46*	tyrosine	3.06,<br>3.21<br>3.95<br>6.90	

										7.20	
11	succinic acid	2.41	s	*29*	glycine	3.56	s	*47*	phenylalanine	3.13,<br>3.29<br>4.00<br>7.33<br>7.39<br>7.42	
12	3-phenylpropionic acid	2.50<br>2.92<br>7.30	m<br>m<br>m	*30*	ethanol	1.19<br>3.66	t<br>q	*48*	histidine	7.09<br>7.87	
13	3-(4-hydroxyphenyl)propionic acid (phloretic acid)	2.45<br>2.82<br>6. 85<br>7.18	t<br>t<br>m<br>m	*31*	glycerol	3.66<br>3.56<br>3.79	m<br>m<br>m	*49*	*N*-acetyl groups	2.06	s
14	phenylacetic acid	3.54<br>7.30<br>7.37	s<br>m<br>m	*32*	propylene glycol	1.15<br>3.88<br>3.55<br>3.45	d	*50*	unknown	1.15<br>3.63	d
15	4-hydroxyphenyl acetic acid	3.45<br>6.87<br>7.17	s<br>m<br>m	*33*	trimethylamine	2.90	s	*51*	unknown	1.15<br>3.73	d
16	δ valerolactam	2.33<br>1.78<br>1.74<br>3.30	t<br>m<br>m<br>t	*34*	dimethylamine	2.73	s	*52*	unknown (acetoin?)	1.38<br>4.43	d
17	formate	8.46	s	*35*	monomethylamine	2.61	s	*53*	unknown	3.16	s
18	ethanolamine	3.15<br>3.83	m<br>m	*36*	putrescine	1.77<br>3.06	m<br>t				

a: ^{1}H multiplicity (s, singlet; d, doublet; dd, doublet of doublets; t, triplet; q, quartet; m, multiplet; sx, sextet)
